# Epidemiology of Pediatric Respiratory Tract Infections During the COVID-19 Era: A Retrospective Multicentric Study of Hospitalized Children in Lebanon Between October 2018 and March 2021

**DOI:** 10.7759/cureus.61669

**Published:** 2024-06-04

**Authors:** Hadi Fakih, Nasab Abdulsater, Zahraa El Hajj Hussein

**Affiliations:** 1 Pediatrics, Faculty of Medical Sciences, Lebanese University, Beirut, LBN; 2 Pediatrics, Sheikh Ragheb Harb University Hospital, Toul, LBN; 3 Pediatrics, Lebanese University, Beirut, LBN

**Keywords:** pre-covid-19, bronchiolitis, lebanon, respiratory tract infection, children, covid-19 eras

## Abstract

Background

The identification of SARS-CoV-2 in December 2019 and its subsequent designation as the causative agent of COVID-19 marked the beginning of an unprecedented global health crisis. As the virus spread rapidly across continents, its impact on various demographic groups, including children, became a subject of intense research. While children were initially thought to be less susceptible to severe COVID-19 illness compared to adults, concerns emerged regarding their vulnerability to other respiratory infections amidst the pandemic. Understanding the epidemiological trends of pediatric respiratory tract infections (RTIs) during the COVID-19 era is crucial for informing public health strategies and clinical management protocols. This study aimed to compare the prevalence and characteristics of pediatric RTIs before and during the COVID-19 pandemic in Lebanon.

Methodology

A retrospective, observational study was conducted by reviewing medical records of children admitted to three tertiary care hospitals in Lebanon: Sheikh Ragheb Harb University Hospital, Al Sahel General University Hospital, and Rafik Al-Hariri University Hospital. Data were collected from October 2018 to March 2021, encompassing both the pre-COVID-19 and COVID-19 eras. A standardized data collection sheet was utilized to gather information on demographic characteristics, clinical presentations, duration of hospitalization, and antibiotic usage.

Results

Our analysis revealed significant shifts in the epidemiology of pediatric RTIs between the pre-COVID-19 and COVID-19 eras. There was a marked decline in the proportion of school-age children hospitalized with RTIs during the pandemic period. However, the overall percentage of Lebanese hospitalized children across different age groups increased significantly during the COVID-19 era. Furthermore, the prevalence of specific RTIs, such as pharyngitis, increased from 1.1% in the pre-COVID-19 to 5.5% during the COVID-19 period (p = 0.016), and the prevalence of bronchiolitis increased from 26.7% to 50.9% (p < 0.001) during the pre-COVID-19 and COVID-19 periods, respectively. This notable rise during the pandemic suggested potential changes in circulating pathogens or diagnostic practices. Importantly, the median length of hospital stays for pediatric RTIs decreased during the COVID-19 era compared to the pre-pandemic period, indicating possible improvements in clinical management or healthcare resource utilization. Analysis of antibiotic usage revealed ceftriaxone as the most frequently prescribed antibiotic in both periods, highlighting its continued relevance in the management of pediatric RTIs.

Conclusions

This study highlights significant epidemiological shifts in pediatric RTIs during the COVID-19 era in Lebanon. These findings underscore the importance of ongoing surveillance and research to adapt public health interventions and clinical practices to evolving infectious disease dynamics. Further investigation is warranted to elucidate the underlying factors driving these changes and optimize strategies for the prevention and management of pediatric RTIs in the context of the ongoing pandemic.

## Introduction

Several studies examining COVID-19 cases and case series in children have been published, shedding light on the clinical manifestations and outcomes of the disease in this demographic. However, the COVID-19 pandemic has also had secondary effects on pediatric healthcare utilization, particularly concerning non-COVID-19-related diseases, which warrant further investigation [1,2].

Reports have documented a decline in consultations for various medical conditions unrelated to COVID-19, coinciding with a significant increase in visits to the emergency room (ED) for symptoms suggestive of COVID-19 infection [3]. For instance, research has shown that the implementation of safety measures, such as mask-wearing and social distancing, has contributed to a decrease in hospitalizations for other respiratory tract diseases among children [4].

A study examining hospitalization trends during the initial phase of the SARS-CoV-2 epidemic found a substantial decrease in overall hospitalizations compared to the same period in previous years. Specifically, there were notable reductions in hospitalizations for bronchiolitis and asthma, which are commonly associated with viral infections and could be influenced by social distancing measures. However, there was no significant change observed in hospitalizations for illnesses not typically linked to viral infections [4].

Moreover, the decline in pediatric hospitalizations observed during the COVID-19 pandemic exceeded the annual reductions reported in previous years, raising concerns about potential delays in seeking necessary medical care amidst the outbreak [4]. The incidence of viral illnesses and outpatient visits for common pediatric infectious disorders has also decreased as a result of social isolation measures, suggesting a potential impact on disease transmission dynamics [5].

Furthermore, a decline in childhood immunization rates and adult hospitalizations for non-COVID-19-related illnesses has been concurrent with the observed decrease in pediatric hospitalizations. These trends have raised concerns about the potential consequences of deferred medical care during the pandemic [6]. Respiratory tract infections (RTIs) represent a significant burden of pediatric morbidity, with community-acquired pneumonia alone accounting for approximately 0.22 episodes per child per year [7]. Among hospitalized children in Lebanon, viral infections were identified as the primary etiology in 70% of RTI cases. The most prevalent viruses included human rhinovirus, respiratory syncytial virus, human bocavirus, human metapneumovirus, and human adenovirus [8]. Additionally, other studies have reported the presence of parainfluenza, echovirus, and coxsackieviruses as causative agents of RTIs in children [9]. These findings underscore the diverse viral etiology of pediatric RTIs and emphasize the importance of targeted prevention and management strategies.

The percentage of RTIs and hospitalizations due to respiratory infections among children during the COVID-19 pandemic has not been studied in Lebanon. Hence, this study was performed to analyze the patterns of change in pediatric hospital admissions during the COVID-19 pandemic compared to the pre-COVID-19 period due to RTI.

## Materials and methods

Ethical considerations

Our study was conducted with adherence to ethical standards, and all necessary approvals were obtained before data collection. Institutional Review Board approval was obtained from the Sheikh Ragheb Harb University Hospital (authorization number: 3279/1/22).

Study design

This study employed a retrospective observational design to investigate the epidemiology of pediatric RTIs in hospitalized children. Data collection was conducted through the review of medical records of children aged between one month and 13 years admitted to three major hospitals in Lebanon. The participating hospitals included Sheikh Ragheb Harb University Hospital located in Toul, Nabatieh Governorate, Al Sahel Hospital in Beirut, and Rafik Hariri University Hospital in Beirut.

The study period spanned from October 2018 to March 2021, allowing for a comparison between the pre-COVID-19 era (October 2018 to February 2020) and the COVID-19 era (February 2020 to March 2021).

Data collection involved a systematic review of medical files and the extraction of relevant information according to a standardized questionnaire. This approach facilitated the collection of comprehensive data on demographic characteristics, clinical presentations, duration of hospitalization, and antibiotic usage among hospitalized children with RTIs.

Study population

We recruited 500 cases of children with RTI between the period of 2018 and 2021.

Inclusion Criteria

We included children between one month and 13 years old admitted with upper or lower RTI with symptoms such as fever, cough, and dyspnea.

Exclusion Criteria

We excluded children with incomplete medical records; children with COVID-19 infection; children hospitalized due to oncological causes; children with chronic diseases such as asthma, cardiovascular disease, and congenital diseases; and children with immune suppression.

Procedures of data collection

After obtaining approval from the hospital’s IRB committee, data were collected from patient’s files regarding all variables of interest. Patients’ data including age, nationality, gender, and geographic region were collected.

Diagnoses of the patients were grouped as follows: (i) Upper respiratory tract infection, including the common cold, tonsillitis, laryngitis, croup, pharyngitis, sinusitis, otitis, and viral rhinitis, and (ii) lower respiratory tract infection, including tracheobronchitis, bronchiolitis, bronchopneumonia, and pneumonia.

Pathogens causing RTIs were detected by polymerase chain reaction (PCR), rapid test, or culture. Treatments administered included antivirals and antibiotics.

Disease characteristics were also collected. These included vital signs, such as systolic blood pressure (SBP), diastolic blood pressure (DBP), heart rate, respiratory rate, oxygen saturation, and fever (temperature), as well as clinical symptoms.

Admission characteristics, including duration of stay in the hospital, intensive care unit need, and oxygen need, were noted.

Laboratory tests included complete blood count, C-reactive protein, urine analysis, and stool analysis. Imaging included chest X-ray (CXR) results and the number of CXRs done. Lastly, co-infections were also noted.

This study design enabled the assessment of temporal trends and differences in the prevalence and characteristics of pediatric RTIs before and during the COVID-19 pandemic, contributing to our understanding of the impact of the pandemic on pediatric respiratory health.

Data analysis

Statistical analysis was conducted using SPSS version 23.0 (IBM Corp., Armonk, NY, USA). Categorical data were expressed as percentages and frequencies, while numerical data were expressed as mean ± standard deviation. The chi-square test was used to study the association between categorical variables. To compare means before and after the pandemic, we used the t-test if the variables had a normal distribution or the Mann-Whitney U test in case of a non-normal distribution of variables. Statistical significance was set at 0.05 and the confidence interval at 95%.

## Results

Study population characteristics and changes in RTI rates

This study included a total of 500 children, among whom 37.2% (n = 186) were females and 62.8% (n = 314) were males. The majority of children were of Lebanese nationality (79.2%, n = 369), with preschoolers comprising the largest age group (67.6%, n = 338), and most children originating from the Nabatieh and Beirut regions (Table 1).

**Table 1:** Demographic characteristics of children with RTIs during pre-COVID-19 and COVID-19 eras. Categorical variables are shown as numbers (n) and percentages (%). n: sample size. RTI = respiratory tract infection

Parameter	Total	During COVID-19 era (n = 55)	Pre-COVID-19 era (n = 445)	P-value
Age (years)				
<3 (preschoolers)	338	47 (85.5)	291 (65.4)	0.003
≥3 (schoolers)	162	8 (14.5)	154 (34.6)	
Nationality				
Lebanese	369	52 (94.5)	317 (71.2)	0.001
Syrian	114	3 (5.5)	111 (24.9)	
Others	17	0 (0.0)	17 (3.8)	
Gender				
Female	186	17 (30.9)	169 (38.0)	0.306
Male	314	38 (69.1)	276 (62.0)	
Geographic region				
Beirut	234	12 (21.8)	222 (49.9)	<0.001
Nabatieh	250	43 (78.2)	207 (46.5)	
Others	16	0 (0.0)	16 (3.6)	

RTI rates

Table 1 illustrates significant differences in RTI rates based on specific demographic characteristics. Notably, the proportion of children aged over three years with RTIs experienced a significant decrease during the COVID-19 era compared to the pre-COVID era (34.6% vs. 14.5%, p = 0.003). Additionally, the percentage of Lebanese children with RTIs significantly increased during the COVID-19 era compared to the pre-COVID-19 era (71.2% vs. 94.5%, p = 0.001), whereas Syrian children exhibited a decrease in RTI rates (24.9% vs. 5.5%) (Table 1). These findings highlight the demographic shifts in RTI rates observed during the different periods examined in this study.

Comparison of pediatric RTI rates pre-COVID-19 vs. COVID-19 era

Table 2 presents a comparison of respiratory infections among children during the COVID-19 pandemic (n = 55) and the pre-COVID-19 period (n = 445). Significant changes in the percentages of children affected by specific respiratory infections were observed.

**Table 2:** Clinical diagnosis of children with RTIs during pre-COVID-19 and COVID-19 eras. RTI = respiratory tract infection

Parameter	Total	During COVID-19 era (n = 55)	Pre-COVID-19 era (n = 445)	P-value
Sinusitis				
No	492	54 (98.2)	438 (98.4)	0.891
Yes	8	1 (1.8)	7 (1.6)	
Tonsillitis				
No	452	52 (94.5)	400 (89.9)	0.269
Yes	48	3 (5.5)	45 (10.1)	
Croup				
No	498	55 (100.0)	443 (99.6)	0.618
Yes	2	0 (0.0)	2 (0.4)	
Pharyngitis				
No	492	52 (94.5)	440 (98.9)	0.016
Yes	8	3 (5.5)	5 (1.1)	
Laryngitis				
No	499	55 (100.0)	444 (99.8)	0.725
Yes	1	0 (0.0)	1 (0.2)	
Common cold				
No	497	55 (100.0)	442 (99.3)	0.541
Yes	3	0 (0.0)	3 (0.7)	
Otitis				
No	495	55 (100.0)	440 (98.9)	0.429
Yes	5	0 (0.0)	5 (1.1)	
Pneumonia				
No	322	42 (76.4)	280 (62.9)	0.050
Yes	178	13 (23.6)	165 (37.1)	
Tracheobronchitis				
No	489	55 (100.0)	434 (97.5)	0.238
Yes	11	0 (0.0)	11 (2.5)	
Bronchopneumonia*				
No	448	52 (96.3)	396 (89.0)	0.094
Yes	51	2 (3.7)	49 (11.0)	
Bronchitis				
No	451	50 (90.9)	401 (90.1)	0.851
Yes	49	5 (9.1)	44 (9.9)	
Bronchiolitis				
No	353	27 (49.1)	326 (73.3)	<0.001
Yes	147	28 (50.9)	174 (26.7)	

There was a notable increase in the percentage of children diagnosed with pharyngitis, increasing from 1.1% to 5.5% (p = 0.016). Similarly, the rate of children diagnosed with bronchiolitis experienced a substantial increase, climbing from 26.7% to 50.9% (p < 0.001) (Table 2). These findings underscore the shifting patterns of respiratory infections among pediatric patients during the COVID-19 pandemic compared to the pre-COVID-19 period.

Comparison of patients’ age in certain RTIs before and during COVID-19

The analysis of our results revealed no significant differences in median age among patients diagnosed with pharyngitis, bronchiolitis, pneumonia, bronchitis, and bronchopneumonia during both the COVID-19 era and the pre-COVID-19 period (p > 0.05) (Table 3).

**Table 3:** Age of patients with respiratory infections before and during COVID-19. *: P-value was obtained using the Mann-Whitney test.

		Median age	P-value*
Pharyngitis	During COVID-19	2.00	0.65
	Pre-COVID-19	0.9	
Bronchiolitis	During COVID-19	0.66	0.56
	Pre-COVID-19	0.58	
Pneumonia	During COVID-19	1.00	0.9
	Pre-COVID-19	1.00	
Bronchitis	During COVID-19	3.00	0.84
	Pre-COVID-19	3	
Bronchopneumonia	During COVID-19	0.53	0.52
	Pre-COVID-19	1.5	

Comparison of vital signs and duration of hospitalization in children with RTI: pre-COVID-19 vs. COVID-19 era

Table 4 compares the vital signs and duration of hospitalization between the pre-COVID-19 era and the COVID-19 pandemic. The data indicate several significant differences in clinical parameters between the two periods.

**Table 4:** Vital signs and duration of hospitalizations of children with RTIs during pre-COVID-19 and COVID-19 eras. *Mann-Whitney U test. SD = standard deviation; RTI = respiratory tract infection

Parameter	Total	During COVID-19 era (n = 55)	Pre-COVID-19 era (n = 445)	P-value
Systolic blood pressure (mmHg) (mean ± SD) median (Q_1_-Q_3_)	101.140 ± 47.188, 100.00 (90.00-110.00)	93.509 ± 10.220, 90.00 (90.00-100.00)	102.083 ± 49.817, 100.00 (90.00-110.00)	<0.001*
Diastolic blood pressure (mmHg) (mean ± SD) median (Q_1_-Q_3_)	55.402 ± 8.918, 56.00 (50.00-60.00)	52.945 ± 8.551, 50.00 (50.00-60.00)	55.706 ± 8.925, 56.00 (50.00-60.00)	0.005*
Heart rate (beats/minute) (mean ± SD) Median (Q_1_-Q_3_)	121.338 ± 17.862, 122.00 (110.00-132.00)	120.436 ± 18.350, 124.00 (118.00-132.00)	121.449 ± 17.819, 122.00 (109.00-132.00)	0.380*
Respiratory rate (mean ± SD) median (Q_1_-Q_3_)	27.498 ± 8.072, 26.00 (24.00-30.00)	28.527 ± 5.088, 28.00 (24.00-31.00)	27.371 ± 8.362, 26.00 (24.00-29.00)	0.007*
O_2_ saturation (%) (mean ± SD) median (Q_1_-Q_3_)	96.803 ± 3.775, 98.00 (96.00-98.00)	96.945 ± 2.527, 98.00 (96.00-98.00)	96.785 ± 3.903, 98.00 (96.00-98.00)	0.704*
Fever (temperature days) (mean ± SD) median (Q_1_-Q_3_)	37.773 ± 2.749, 37.50 (37.00-38.10)	37.344 ± 0.885, 37.00 (36.90-37.90)	37.826 ± 2.893, 37.60 (37.00-38.15)	0.001*
Duration of hospitalization (days) (mean ± SD) median (Q1-Q3)	6.358 ± 6.288, 4.00 (3.00-7.00)	4.073 ± 1.709, 3.00 (3.00-5.00)	6.640 ± 6.585, 5.00 (3.00-7.00)	<0.001*

Children hospitalized during the COVID-19 pandemic exhibited lower SBP (p < 0.001), lower DBP (p = 0.005), and lower fever (p = 0.001) compared to the pre-COVID-19 era. Additionally, the duration of hospitalization was significantly shorter during the COVID-19 era (p < 0.001). However, children with respiratory infections during the COVID-19 pandemic had a higher respiratory rate compared to the pre-COVID-19 period (p = 0.007).

Table 5 displays the analysis of laboratory data, including basophils and hemoglobin levels, in children with respiratory infections during the pre-COVID-19 and COVID-19 periods. The results indicate that basophils and hemoglobin levels were significantly lower in children during the COVID-19 era (p = 0.004 and p < 0.001, respectively). However, no significant differences were observed in the other laboratory findings between the pre-COVID-19 and COVID-19 eras. Additionally, concerning urine analysis and stool tests, the percentage of positive cases did not show a significant change during the COVID-19 pandemic.

**Table 5:** Laboratory data, urine analysis, and stool test of children with RTIs during the pre-COVID-19 and COVID-19 eras. Categorical variables are shown as numbers (n) and percentages (%). *: Mann-Whitney U test. RTI = respiratory tract infection; WBCs = white blood cells; Hb = hemoglobin; HCT = hematocrit; CRP = C-reactive protein; Neut = neutrophils; Lymphs = lymphocytes; Baso = basophils; PLT = platelets; SD = standard deviation

Parameter	Total	During COVID-19 era (n = 55)	Pre-COVID-19 era (n = 445)	P-value
Laboratory findings				
WBCs (×10^3^ per mm^3^) (mean ± SD) median (Q_1_-Q_3_)	12.806 ± 6.032, 11.80 (8.60-15.42)	13.908 ± 6.809, 11.86 (8.68-17.00)	12.670 ± 5.924, 11.80 (5.58-15.25)	0.280*
Neut (in %) (mean ± SD) median (Q_1_-Q_3_)	54.971 ± 20.482, 56.50 (39.00-71.50)	50.629 ± 18.761, 54.60 (35.50-65.00)	55.509 ± 20.641, 56.90 (40.00-71.85)	0.079*
Lymphs (in %) (mean ± SD) median (Q_1_-Q_3_)	34.402 ± 25.151, 32.10 (20.90-44.82)	35.645 ± 17.577, 33.20 (23.00-46.80)	34.249 ± 25.946, 32.00 (20.90-44.15)	0.378*
Baso (in %) (mean ± SD) median (Q_1_-Q_3_)	0.633 ± 0.574, 0.40 (0.20-0.90)	0.408 ± 0.339, 0.30 (0.20-0.49)	0.661 ± 0.591, 0.43 (0.20-0.94)	0.004*
Hb (g/dL) (mean ± SD) median (Q_1_-Q_3_)	8.838 ± 3.974, 10.10 (4.60-12.20)	6.674 ± 3.548, 4.60 (4.00-10.60)	9.105 ± 3.946, 10.40 (4.79-12.30)	<0.001*
HCT (in %) (mean ± SD) median (Q_1_-Q_3_)	33.343 ± 4.495, 33.40 (31.00-36.00)	32.702 ± 3.829, 32.80 (30.00-35.00)	33.423 ± 4.568, 33.80 (31.00-36.00)	0.117*
CRP (mg/dL) (mean ± SD) median (Q_1_-Q_3_)	46.115 ± 64.635, 20.00 (7.42-60.00)	45.460 ± 64.194, 15.70 (7.80-51.20)	46.195 ± 64.761, 21.00 (7.20-60.95)	0.832*
PLT (×10^3^/μL) (mean ± SD) median (Q_1_-Q_3_)	370.859 ± 139.400, 353.00 (139.40-453.75)	374.145 ± 127.124, 353.00 (300.00-423.00)	370.454 ± 140.970, 353.00 (275.00-455.50)	0.824*
Urine analysis				
Negative	326	32 (91.4)	294 (90.5)	0.853
Positive	34	3 (8.6)	31 (9.5)	
Stool test				
Negative	116	18 (94.7)	98 (96.1)	0.787
Positive	5	1 (5.3)	4 (3.9)	

Table 6 presents a comparative analysis of the clinical presentation of children with RTI between the pre-COVID-19 and COVID-19 pandemic. Notably, the percentage of children experiencing dyspnea significantly increased from 13.9% to 40% during the COVID-19 era (p < 0.001). However, no significant associations were observed between the other clinical presentations and the pre-COVID-19 and COVID-19 pandemic. These findings highlight the specific impact of the COVID-19 pandemic on the respiratory symptoms of pediatric RTI cases, with a substantial increase in dyspnea observed during the COVID-19 era.

**Table 6:** Clinical presentation of children with RTIs during the pre-COVID-19 and COVID-19 eras. Categorical variables are shown as numbers (n) and percentages (%). RTI = respiratory tract infection

Parameter	Total	During COVID-19 era (n = 55)	Pre-COVID-19 era (n = 445)	P-value
Cough				
No	94	9 (16.4)	85 (19.1)	0.624
Yes	406	46 (83.6)	360 (80.9)	
Rhinorrhea				
No	346	39 (70.9)	307 (69.0)	0.771
Yes	154	16 (29.1)	138 (31.0)	
Dyspnea				
No	416	33 (60.0)	383 (86.1)	<0.001*
Yes	84	22 (40.0)	62 (13.9)	
Fever				
No	128	14 (25.5)	114 (25.6)	0.979
Yes	372	41 (74.5)	331 (74.4)	
Duration of fever (days) (mean ± SD), median (Q_1_-Q_3_)	2.012 ± 1.829, 2.00 (0.00-3.00)	1.927 ± 1.730, 2.00 (0.00-3.00)	2.022 ± 1.843, 2.00 (0.00-3.00)	0.828*

Logistic regression analysis of children with RTI: pre-COVID-19 vs. COVID-19 era

The analysis revealed that during the COVID-19 era, children were 6.814 times more prone to have pharyngitis than in the pre-COVID-19 era (CI = 1.410-32.936; p = 0.017). Additionally, children were 2.284 times more likely to have bronchiolitis during the COVID-19 era (CI = 1.027-5.081; p < 0.043). These findings provide evidence of significant associations between the COVID-19 era and the increased likelihood of pharyngitis and bronchiolitis among children with RTI.

Antibiotic use

According to the data, during the COVID-19 era, 21.80% of children with RTI received antibiotics, while in the pre-COVID-19 era, 22.70% of children received antibiotics. The majority of children, both during the COVID-19 era (78.20%) and pre-COVID-19 era (77.30%), did not receive antibiotics for their RTI. This information provides insights into the utilization of antibiotics in the management of pediatric RTI cases, highlighting that there was no significant difference in antibiotic use between the two periods.

The analysis of antibiotic usage among children with RTI revealed consistent trends in prescription patterns during the pre-COVID-19 and COVID-19 eras. Ceftriaxone emerged as the most frequently prescribed antibiotic in both periods, accounting for 26.7% and 23.6% of cases, respectively.

Seasonal variation of pneumonia and bronchiolitis pre-COVID-19 and during the COVID-19 era

In our study comparing the seasonal variation of pneumonia and bronchiolitis before and during the COVID-19 era, we observed distinct patterns. In the pre-COVID-19 era (2018-2019), bronchiolitis cases were prominent in January 2019, aligning with the typical winter season, while pneumonia cases peaked in April 2019, corresponding to the spring season.

During the COVID-19 era, a notable shift occurred in the temporal distribution of both bronchiolitis and pneumonia. In March 2020, which is traditionally a spring month, we observed peaks for both respiratory conditions. However, in the winter of 2020-2021, there were no notable peaks in bronchiolitis and pneumonia cases.

It is important to note that the data collection spanned two winter seasons before the onset of the COVID-19 pandemic and one winter season during the pandemic.

## Discussion

This study provides valuable insights into the epidemiology and clinical characteristics of RTIs in children during the COVID-19 pandemic compared to the pre-COVID-19 period. We observed significant shifts in RTI rates and demographic patterns, shedding light on the multifaceted impact of the pandemic and associated preventive measures.

The decline in hospital admissions for RTIs among school-age children during the COVID-19 era aligns with findings from other studies, indicating a reduction in overall RTI burden among this age group [10]. However, the higher proportion of preschoolers admitted to hospitals during both periods underscores the vulnerability of younger children to RTIs, possibly due to factors such as immature immune systems and increased exposure in daycare settings [10,11].

Notably, this study revealed a substantial increase in the proportion of children diagnosed with pharyngitis and bronchiolitis during the COVID-19 era. Logistic regression analysis further highlighted the heightened susceptibility to bronchiolitis and pharyngitis during this period, suggesting a possible association with the COVID-19 pandemic [12,13]. These findings echo reports from other regions, indicating shifts in respiratory illness patterns and increased severity among certain pediatric populations [12,14].

Clinical assessments revealed significant differences in vital signs and laboratory parameters between the pre-COVID-19 and COVID-19 periods. Children hospitalized during the COVID-19 era exhibited lower blood pressure, higher respiratory rates, and decreased hemoglobin levels, indicating potential variations in disease severity and clinical presentation [15,16]. The notable increase in dyspnea among pediatric patients during the COVID-19 era underscores the importance of vigilance for respiratory distress, particularly in bronchiolitis cases [16].

Contrary to expectations, our study did not find substantial changes in the need for oxygen and nebulizer treatments during the COVID-19 period, suggesting consistent respiratory support requirements despite the pandemic. However, variations in treatment patterns were observed, with certain antibiotics being more commonly prescribed during the COVID-19 era, highlighting the importance of prudent antibiotic stewardship [17,18].

Seasonal variation analysis revealed shifts in the temporal distribution of pneumonia and bronchiolitis during the COVID-19 pandemic, with peaks observed in atypical months. These findings suggest potential disruptions to traditional seasonal patterns of respiratory infections, possibly influenced by pandemic-related factors such as altered social behaviors and infection control measures [19,20].

Despite providing valuable insights, our study has several limitations. Data collection constraints, including missing variables such as bacterial cultures and viral panels, may have impacted the accuracy of our analyses. Additionally, the absence of information on co-infections limits our understanding of the full spectrum of respiratory pathogens circulating during the study periods.

## Conclusions

This study provides valuable insights into the shifting landscape of RTIs in children before and during the COVID-19 pandemic. it highlights significant changes in the patterns of respiratory illnesses, with a notable rise in bronchiolitis cases that were hospitalized during the COVID-19 era, This change underscores the policy of admitting only severe cases of RTI during the pandemic era. Moreover, antibiotic prescription patterns remained unchanged across both periods. These findings underscore the importance of judicious antibiotic use, the ongoing efforts to optimize treatment strategies, and the importance of using more rapid and accurate diagnostic tests such as PCR.

This study contributes to a better understanding of the impact of the COVID-19 pandemic on pediatric RTIs, informing future research directions and public health interventions aimed at mitigating disease burden and optimizing patient outcomes. Continued surveillance and adaptive strategies will be crucial as we navigate the post-pandemic landscape of respiratory infections in children.
